# Blood cells and hematological parameters of the mountain dragon, *Diploderma micangshanensis* (Squamata: Lacertilia: Agamidae)

**DOI:** 10.7717/peerj.12397

**Published:** 2021-10-26

**Authors:** Yinlong Bai, Guanglu Li, Shuaichao Lin, Jianli Xiong

**Affiliations:** 1 College of Animal Science and Technology, Henan University of Science and Technology, Luoyang, Henan Province, China; 2 Ecological Security and Protection Key Laboratory of Sichuan Province, Mianyang Normal University, Mianyang, Sichuan Province, China

**Keywords:** Adaptive evolution, Erythrocyte, Hematology, Mountain dragon, Sexual differences

## Abstract

Hematological characteristics reflect the health status of animals and their physiological adaptation to the environment. However, few studies focused on the species of *Diploderma*. In this study, the blood cells and the hematological parameters of *Diploderma micangshanensis*, a species endemic to China, were examined based on 48 healthy adult (32 males and 16 females). The blood cells and hematological parameters of *D. micangshanensis* were similar to those of other lizard species. Although the values of erythrocyte morphometric characters and hematological parameters varied between males and females, the differences were only significant in the case of the hematocrit and erythrocyte size, which may allow for higher oxygen availability in males. Hemoglobin, mean corpuscular hemoglobin concentration, and mean corpuscle volume were strongly affected by the snout-vent length and/or body mass, which reflect the physiological adaptation to the oxygen requirement of different individuals. This is the first report of hematological data from a species of *Diploderma*, and the results will provide data for research on the adaptive evolution and health assessment in this species and other congeners.

## Introduction

Blood transports nutrients, oxygen, and cellular metabolic waste (Vernberg, 1955). The hematological characteristics within a species change with variations in animal physiology (*e.g.*, reproductive status), sex, and the external environment (*e.g.*, temperature, season, and stress) (Eatwell, Hedley & Barron, 2014); thus, these characteristics reflect the health status of animals and their physiological adaptation to the environment (Hidalgo-Vila et al., 2007; Davis & Davis, 2008). The hematological characteristics of lizards have been widely reported, such as those of *Gallotia simonyi*, *G. bravoana*, *G. intermedia* (Silvestre et al., 2004), *Tupinambis merianae* (Troiano, Gould & Gould, 2008), *Laudakia caucasia*, *L. stellio*, *Phrynocephalus horvathi*, *Trapelus lessonae* (Gül & Tosunoğlu, 2011), *Eutropis carinata* (Parida, Dutta & Pal, 2013), *P. erythrurus* and *P. przewalskii* (Lu et al., 2015), *Takydromus amurensis*, *Eremias argus*, *Lacerta agilis*, *Zootoca vivipara* (Lin et al., 2015), and *Phrynosoma cornutum* (McEntire et al., 2018). These studies have demonstrated that hematological characteristics change with species, the environment, and physiological status. These results also provide important data for the research on the adaptive evolution and health assessment of lizards. For example, the differences of hemoglobin concentration (Hb), hematocrit (Hct), erythrocyte mean cell volume (MCV), mean cell hemoglobin concentration (MCHC) value among *Laudakia caucasia*, *L. stellio*, *P. horvathi* and *Trapelus lessonae* were explained by the differences of activity level during the day (Gül & Tosunoğlu, 2011). Compared with *P. przewalskii* (inhabits in lower altitude), *P. erythrurus* (living in higher altitude) increased red blood cell count (RBC), Hb and Hct to adapt high-altitude hypoxia environment (Lu et al., 2015).

*Diploderma micangshanensis* is a lizard species endemic to China. This species was described by Song (1987) based on specimens collected from Qingmuchuan, Ningqiang County, Shaanxi Province, China. Since then, *D. micangshanensis* has been discovered in Huayin, Luonan, Shangnan, Shanyang, Zhashui, and Foping in Shaanxi Province (Zhao, Zhao & Zhou, 1999); Wenxian, Kangxian, and Wudu in Gansu Province (Li, Li & Wang, 2000; Wang et al., 2019a); Jiuzhaigou and Guangyuan in Sichuan Province; Shiyan in Hubei Province (Gao & Qin, 2000; Wang et al., 2019a); and Xixia and Jiyuan in Henna Province (Zhao, Wang & Lu, 2012). *D. micangshanensis* inhabits the forest margin of coniferous and broad-leaved mixed forests or broad-leaved forests (Wang et al., 2019a). This species was listed as a species of least concerned species on the Red List of China’s Vertebrates (Jiang et al., 2016). However, only the taxonomy, distribution, and phylogeny (Wang et al., 2019a; Wang et al., 2019b; Li et al., 2021) of this species have been reported.

In August 2018, some *Diploderma* specimens were discovered in Luoning County, Luoyang, Henan Province, and identified as *D. micangshanensis* based on morphological characteristics, and this result was confirmed by a molecular study (Li et al., 2021). These specimens provided a chance to examine the hematological characteristics of this species. Thus, the blood cells and hematological parameters of *D. micangshanensis* were examined in the present study. The aims of this study are to (1) describe the characteristics of blood cells and hematological parameters; (2) compare the hematological characteristics between sexes and determine if there are sexual differences; and (3) determine if the snout-vent length (SVL) and body mass affect the hematological characteristics. This study will provide data for health monitoring and assessment of this species, and will also help with research on the adaptive evolution of this species.

## Material and Methods

Forty-eight healthy adult *D. micangshanensis* (32 males and 16 females) were used in this study. The specimens were collected by hands from Luoning County, Henan Province, China (34°16′48″N, 111°43′5″E) in August 2018. Upon arrival in the laboratory, the animals were anesthetized in 0.045% solution of MS-222 (Yu’anbao, Shandong Jinjiang Shui’an Biotechnology Co Ltd). When the animals were motionless, they were removed out from MS-222 solution. The body mass and SVL of each anesthetized individual were measured using an electronic balance and digital calipers, respectively. The individual was fixed on a foam board, heart were exposed, and blood samples were collected by cardiac ventricular puncture *via* heparinized hematocrit capillaries. And then animals were killed by ethanol anesthesia, sexual maturity was determined by gonadal development. Voucher specimens were stored in 10% formalin and deposited in the Museum of Henan University of Science and Technology.

Blood smears were prepared using the push slide technique to examine erythrocyte morphometry. Dried blood smears were stained with Wright’s stain and examined under a light microscope (OLYMPUS CX31; Tokyo, Japan). The red blood cell count, leukocyte count, as well as the erythrocyte morphometry were determined by the methods used in Xiong et al. (2018). A total of 100 leukocytes were counted in the blood smear of each individual for differential leukocyte counts, which were manually performed under a light microscope. Then, the percentage of each leukocyte type for each individual were calculated according to the observed data. Hb were measured with Sahil’s haemometer. Hct was determined by standard centrifugation (Xiangzhi Centrifuge TG12, Changsha Xiangzhi Centrifuge Instrument Co Ltd) in microhematocrit tubes with 12,000 revolutions per minute for 5 min. Hematocrit was calculated from the proportion of the blood cell volume in the total blood volume. MCV, mean cell hemoglobin (MCH), and MCHC were calculated according to Wintrobe’s formula (Wintrobe, 1933).

All data were tested for normality using the Kolmogorov–Smirnov test and homogeneity of variance was determined by Levene’s test. As all data were normal and homogeneous, a one-way analysis of variance (ANOVA) was used to test for the effect of sex. Linear regression was used to determine the relationships between SVL, body mass, erythrocyte morphometry, and the hematological parameters. All statistical analyses were carried out with SPSS software, version 22.0 (SPSS Inc., Chicago, IL, USA). Values are presented as mean ± standard error, and a *p*-value < 0.05 was considered significant.

This research complies with the laws and ethical standards of China. All experiments were conducted in accordance with protocols were approved by the Animal Care and Use Committee of the College of Animal Science and Technology, Henan University of Science and Technology (HNUST-CAST201808001).

## Result

**Figure 1:**
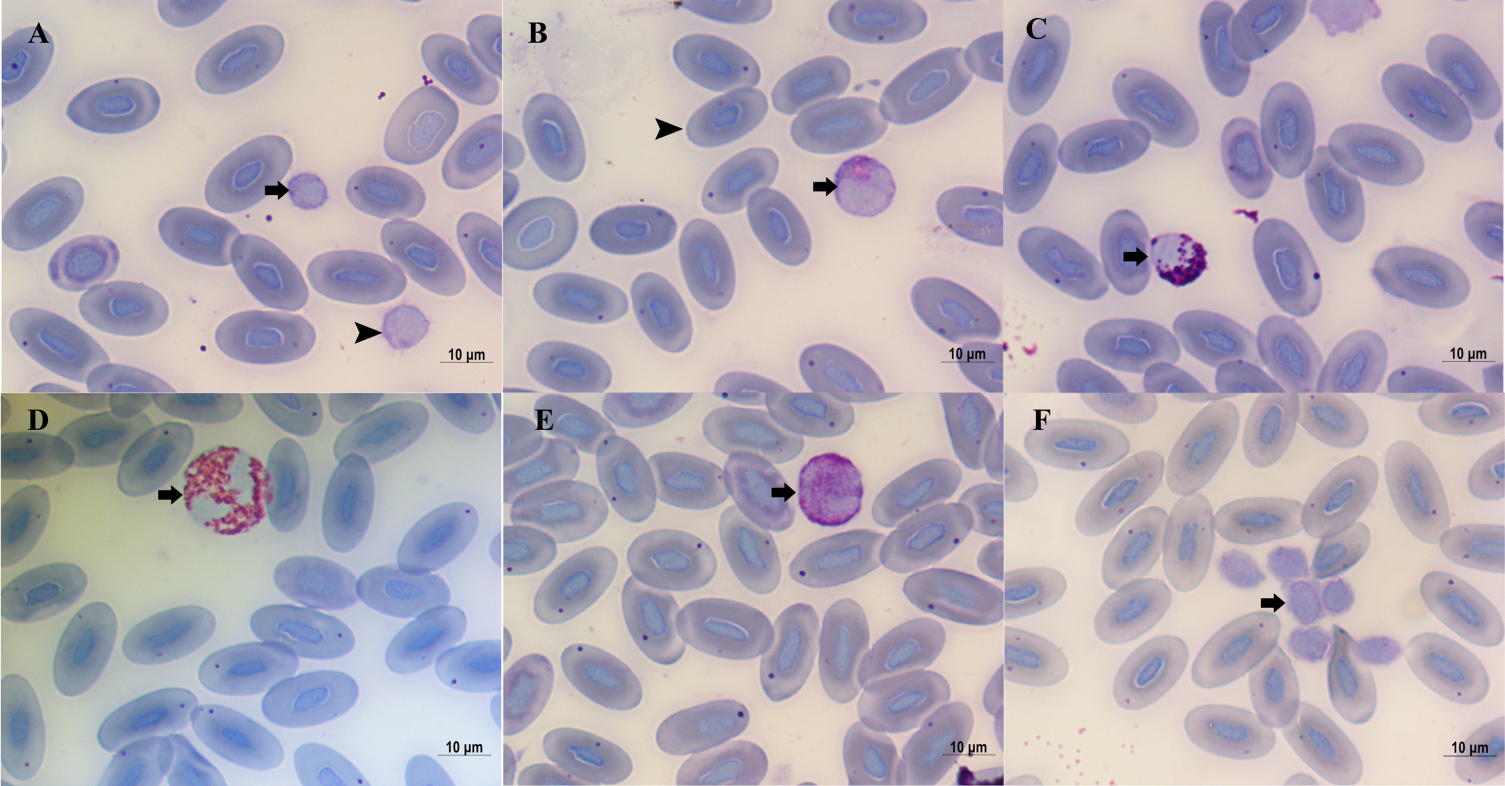
Blood cells of *Diploderma micangshanensis*. (A) Large (arrowhead) and small (arrow) Lymphocyte, (B) Monocyte (arrow) and erythrocyte (arrowhead), (C) Eosinophil (arrow), (D) Heterophil (arrow), (E) Basophil (arrow), (F) thrombocyte (arrow).

Blood cells are depicted in Fig. 1. Erythrocytes are oval cells with an ellipsoidal nucleus located at the center of the erythrocyte (Fig. 1). The leukocytes were divided into lymphocytes, monocytes, eosinophils, basophils, and heterophils according to their morphological characteristics. Lymphocytes, the most abundant leukocytes in blood smears (Table 1), were round, contained small amount of cytoplasm. The cytoplasm was stained dark purple, and the nucleus was stained dark purplish blue. Two different-sized lymphocytes were found (Fig. 1A). The mean diameter of large lymphocytes was 8.85 µm (8.03–9.89 µm) and of small lymphocytes was 7.06 µm (5.66–7.96 µm). Monocytes, the second most abundant leukocytes, had a kidney or horseshoe-shaped nucleus. The cytoplasm was stained reddish-brown, whereas the nuclei stained dark purplish-blue (Fig. 1B). Monocytes had a mean diameter of 11.38 µm (8.39–14.35 µm). Eosinophils, the third most abundant leukocytes, were spherical, characterized by stained reddish granules and nucleus were stained dark blue (Fig. 1C). The mean diameter of the eosinophils was 10.47 µm (8.28–13.08 µm). Heterophils, the fourth most abundant leukocytes, were spherical with a segmented nucleus. The cytoplasm was stained reddish-brown, and the nucleus was stained dark purplish-blue (Fig. 1D). The mean diameter of neutrophils was 15.81 µm (11.11–19.58 µm). Basophils, the scarcest leukocytes, were characterized by the presence of round lightly basophilic granules of various sizes in the cytoplasm (Fig. 1E). The diameter of basophils was 12.59 µm (9.67–16.82 µm). The thrombocytes were small and irregular in shape, and clumped together in blood smear (Fig. 1F). The percentage of monocytes, eosinophils, and basophils was higher in females than in males, however, the percentage of lymphocytes and neutrophils was higher in males than in females. The difference was insignificant for all percentage of leukocytes (Table 1).

**Table 1:** Leukocyte count observed in *Diploderma micangshanensis*, and the results of comparison between males and females.

Parameters	Sex	Mean ± SE	Range	Sig
	♀	72.79 ± 1.69	62∼84	
Lymphocytes (%)	♂	73.25 ± 1.28	56∼87	*p* = 0.833
	♀♂	73.10 ± 1.01	62∼87	
	♀	10.32 ± 0.80	5∼15	
Monocytes (%)	♂	9.59 ± 0.70	3∼17	*p* = 0.527
	♀♂	9.83 ± 0.54	1∼17	
	♀	4.30 ± 0.61	1∼10	
Heterophils (%)	♂	5.75 ± 0.56	1∼12	*p* = 0.115
	♀♂	5.27 ± 0.43	1∼12	
	♀	7.75 ± 0.79	4∼16	
Eosinophils (%)	♂	6.97 ± 0.45	3∼12	*p* = 0.370
	♀♂	7.23 ± 0.41	3∼16	
	♀	4.84 ± 0.56	1∼9	
Basophils (%)	♂	4.44 ± 0.29	1∼9	*p* = 0.488
	♀♂	4.57 ± 0.27	1∼9	

The erythrocyte morphometric data are summarized in Table 2. The mean values of all erythrocyte characteristics in females were larger than those in males; however, one-way ANOVA revealed that only EL (*p* = 0.04) and EA (*p* = 0.03) were significantly different between the sexes.

**Table 2:** Erythrocytes morphometry in *Diploderma micangshanensis*, and the results of comparison between males and females.

Characters	Sex	Mean ± SE	Range	Sig
	♀	19.12 ± 0.16	17.77∼20.10	
Erythrocyte length (EL, μm)	♂	18.71 ± 0.11	17.33∼19.95	*P* = 0.04
	♀♂	18.85 ± 0.10	17.33∼20.10	
	♀	10.58 ± 0.16	9.81∼11.70	
Erythrocyte width (EW, μm)	♂	10.17 ± 0.12	8.94∼11.99	*P* = 0.05
	♀♂	10.30 ± 0.10	8.94∼11.99	
	♀	158.94 ± 3.50	140.68∼179.57	
Erythrocyte area (EA, μm^2^)	♂	149.53 ± 2.40	125.99∼187.72	*P* = 0.03
	♀♂	152.67 ± 2.06	125.99∼187.72	
	♀	7.65 ± 0.15	6.74∼8.89	
Nucleus length (NL, μm)	♂	7.55 ± 0.09	6.40∼8.39	*P* = 0.539
	♀♂	7.58 ± 0.07	6.40∼8.90	
	♀	3.72 ± 0.09	3.18∼4.18	
Nucleus width (NW, μm)	♂	3.62 ± 0.05	2.99∼4.36	*P* = 0.315
	♀♂	3.65 ± 0.05	2.99∼4.36	
	♀	22.39 ± 0.85	17.88∼28.80	
Nucleus area (NA, μm^2^)	♂	21.52 ± 0.52	16.15∼27.68	*P* = 0.363
	♀♂	21.81 ± 0.45	16.15∼28.80	

The hematological parameters are shown in Table 3. Males had larger mean values than females for all hematological parameters, except MCHC, but only Hct was significantly different (*p* = 0.03) between the sexes.

**Table 3:** Hematological parameters observed in *Diploderma micangshanensis*, and the results of comparison between males and females.

Parameters	Sex	Mean ± SE	Range	Sig
	♀	4.92 ± 0.17	3.80∼6.20	
Hemoglobin (g/dl)	♂	5.31 ± 0.14	3.30∼7.00	*P* = 0.105
	♀♂	5.18 ± 0.11	3.30∼7.00	
	♀	5.11 ± 0.19	3.70∼6.27	
Erythrocyte count (10^5^/mm^3^)	♂	5.35 ± 0.19	3.15∼7.40	*P* = 0.416
	♀♂	5.27 ± 0.14	3.15∼7.40	
	♀	2.93 ± 0.27	1.56∼5.61	
Leukocyte count (10^4^/mm^3^)	♂	3.28 ± 0.24	1.41∼6.49	*P* = 0.372
	♀♂	3.17 ± 0.18	1.41∼6.49	
	♀	27.12 ± 1.05	20.19∼39.07	
Hematocrit (%)	♂	30.71 ± 1.00	19.56∼43.30	*P* = 0.030
	♀♂	29.51 ± 0.79	19.56∼43.30	
	♀	5.40 ± 0.27	4.10∼7.50	
Mean cell volume (10^2^/fl)	♂	5.88 ± 0.23	3.90∼8.70	*P* = 0.208
	♀♂	5.72 ± 0.18	3.90∼8.70	
	♀	9.71 ± 0. 32	7.59∼12.61	
Mean cell hemoglobin (pg)	♂	10.17 ± 0.35	6.76∼14.29	*P* = 0.404
	♀♂	10.02 ± 0.25	6.76∼14.29	
	♀	18.49 ± 0.91	13.10∼25.00	
Mean cell hemoglobin concentration (%)	♂	17.59 ± 0.50	12.30∼25.40	*P* = 0.349
	♀♂	17.89 ± 0.45	12.30∼25.40	

The mean SVL values were 58.76 ± 1.25 mm (range 49.47–65.68) in females, and 59.45 ± 0.75 mm (range 50.70–64.45) in males, respectively. The mean body mass values were 7.54 ± 0.44 g (range 4.26–10.71) in females and 7.90 ± 0.32 g (ranges 4.06–10.13) in males, respectively. The linear regression analysis results of erythrocyte morphometry, the hematological parameters, SVL, and body mass are shown in Table 4. The Hb and MCHC values were affected by SVL (Hb: *p* = 0.011, MCHC: *p* = 0.006) and body mass (Hb: *p* = 0.017, MCHC: *p* = 0.012); however, only the MCV value was significantly affected by body mass (*p* = 0.039).

**Table 4:** Regression results of erythrocyte morphometry, hematological parameters and SVL, body mass in *Diploderma micangshanensis*.

Characters	SVL	Body mass
Erythrocyte length	F_1,51_ = 1.298, *P* = 0.260	F_1,51_ = 0.756, *P* = 0.389
Erythrocyte width	F_1,51_ = 0.043, *P* = 0.837	F_1,51_ = 0.355, *P* = 0.554
Erythrocyte size	F_1,51_ = 0.330, *P* = 0.568	F_1,51_ = 0.576, *P* = 0.452
Nucleus length	F_1,51_ = 0.871, *P* = 0.356	F_1,51_ = 1.104, *P* = 0.299
Nucleus width	F_1,51_ = 3.016, *P* = 0.089	F_1,51_ = 3.527, *P* = 0.067
Nucleus size	F_1,51_ = 2.215, *P* = 0.143	F_1,51_ = 2.662, *P* = 0.110
Hemoglobin	F_1,51_ = 7.078, *P* = 0.011	F_1,51_ = 6.194, *P* = 0.017
Erythrocyte count	F_1,51_ = 1.947, *P* = 0.170	F_1,51_ = 2.722, *P* = 0.106
Leucocyte count	F_1,51_ = 0.012, *P* = 0.915	F_1,51_ = 0.006, *P* = 0.938
Hct	F_1,51_ = 0.213, *P* = 0.647	F_1,51_ = 0.089, *P* = 0.767
MCV	F_1,51_ = 3.946, *P* = 0.053	F_1,51_ = 4.510, *P* = 0.039
MCH	F_1,51_ = 0.020, *P* = 0.889	F_1,51_ = 0.030, *P* = 0.863
MCHC	F_1,51_ = 8.481, *P* = 0.006	F_1,51_ = 6.852, *P* = 0.012

## Discussion

Up to now, 24 species have been recognized in the genus *Diploderma*, and most (23 species) are distributed in China (Wang et al., 2019a). The hematological characteristics of these species have not been examined. This is the first study on the hematological characteristics of a species in this genus. In lizards the mean value of lymphocytes were 54.9%, heterophils were 25.7%, eosinophils were 5.8%, basophils were 6.6%, and monocytes were 5.9%, respectively (Davis, 2009). In this study, lymphocytes were the dominant leucocytes, followed by monocytes, eosinophils, heterophils, and basophils. Arikan & Çiçek (2014) reported that the EL, EW, erythrocyte size, NL, NW, and nuclear size ranges were 12.83–16.85 μm, 7.97–10.75 μm, 100.78–120.71 μm, 6.12–7.84 μm, 4.0–5.1 μm, and 24.76–35.42 μm, respectively, in species of the family Agamidae. *D. micangshanensis* had a relatively larger EL and erythrocyte size, and a smaller NL and nuclear size compared to these results. Eatwell, Hedley & Barron (2014) reported that Hb values ranged from 5.5 to 12 g/dl, erythrocyte count was 5 ×10^5^ /mm^3^ to 2 ×10^6^/mm^3^, Hct was 20% to 45%, MCV was 2 ×10^2^ fl to 1.2 ×10^3^ fl, MCH was 6 pg to 10 pg, and MCHC was 22% to 41% in reptilian species. Most of the hematological parameters of *D. micangshanensis* were consistent with these results except MCHC, which was lower than the results of Eatwell, Hedley & Barron (2014). These differences in blood cells and hematological parameters demonstrate that hematological characteristics vary among species.

Sexual difference is a common phenomenon in animal kingdom, which occur in morphology, behavior, and physiology. Sexual differences in hematological characteristics have been discovered in some lizards, such as Hct in the mainland population of *Holbrookia propinqua* (Judd, 1974), Hb and MCH in *L. stellio*, and Hb in *L. caucasia* (Gül & Tosunoğlu, 2011), basophil counts in *P. cornutum* (McEntire et al., 2018), EL and EW, NW, and erythrocyte and leukocyte counts in *Saara loricata* (Takesh et al., 2020). In the present study, EL, EA, and Hct of *D. micangshanensis* were significantly different between the sexes. Males had smaller erythrocytes size and a higher Hct value. Erythrocyte size reflects the capacity to carry oxygen and carbon dioxide, with smaller erythrocytes having relatively larger surface areas and a higher carrying capacity (Xiong et al., 2017). Hct refers to the ratio of the volume of erythrocytes to the total volume of blood after separating the erythrocytes from the plasma by centrifugation. Thus, Hct also reflects the capacity to carry oxygen and carbon dioxide, and a higher Hct increases oxygen transport capacity (Lu et al., 2015; González-Morales et al., 2017). A high Hct is realized by an increase in the erythrocyte count or increased erythrocyte size (Judd, 1974; González-Morales et al., 2017). Here, male *D. micangshanensis* had a higher Hct, which may have been contributed by the erythrocyte count, because males have smaller erythrocytes, and a higher erythrocyte count (Tables 1 and 2). Until now, there is no report on the reproductive biology of *D. micangshanensis*, but typical reproductive behaviors of lizards were observed during the field collection of specimens in the present study. Males shook their tails and pushed their heads and their bodies up in front of females, and females kept quiet (personal observation). The sexual differences in erythrocyte size and Hct maybe an advantage for higher oxygen availability in males as needed for these behavioral differences.

SVL and body mass are two important characteristics that can affect respiration, metabolism, and digestion. Body size and body mass also affect hematological characteristics. For example, the dimensions of erythrocytes in *Ambystoma talpoideum* (Davis & Davis, 2008) and *Plethodon cicnereus* (Maerz et al., 2009) increase with body size. The PCV of *Agkistrodon piscivorus* is positively correlated with SVL and body mass (Minter et al., 2013). Here, Hb and MCHC of *D. micangshanensis* were positively correlated with SVL and body mass, whereas MCV was negatively correlated with body mass (Table 3). As the oxygen requirement is positively correlated with SVL and body mass (Feder, 1976; Feder, 1977), a higher Hb and MCHC increase oxygen transport capacity, and a lower MCV reduces the consumption of oxygen by erythrocytes and results in better transport of oxygen. Thus, the correlations between SVL, body mass and Hb, and MCHC and MCV reflect the physiological adaptation to the oxygen requirement of different individuals.

This is the first report on the hematological data of *D. micangshanensis*. The blood cells and hematological parameters were consistent with other lizards. Significant sexual differences in Hct and erythrocyte size were observed, and Hb, MCHC, and MCV were strongly affected by SVL and/or body mass. These results will provide data for research on the adaptive evolution and health assessment of this species.

##  Supplemental Information

10.7717/peerj.12397/supp-1 Supplemental Information 1 The raw data of Diploderma micangshanensis All specimen information and erythocyte morphmetry and hematological parameters of each specimens.

## Additional Information and Declarations

### Field Study Permissions

The following information was supplied relating to field study approvals (i.e., approving body and any reference numbers): Xinjie Dai of Shenlingzhai national Forest Park.

### Data Availability

The following information was supplied regarding data availability: The raw data of *Diploderma micangshanensis* are available in the Supplemental File.
